# Study on Quasi-Open Microwave Cavity Sensor Measuring Pulverized Coal Mass Concentration in Primary Air Pipe

**DOI:** 10.3390/s25123657

**Published:** 2025-06-11

**Authors:** Yiguang Yang, Lianyong Zhang, Chenlong Wang, Lijun Chen, Hao Xu, Shihao Song

**Affiliations:** 1 School of Automation Engineering, Northeast Electric Power University, Jilin 132011, China; yyguang@neepu.edu.cn (Y.Y.); 2202300771@neepu.edu.cn (L.Z.); 2202300721@neepu.edu.cn (C.W.); 2202401274@neepu.edu.cn (S.S.); 2 Guoneng Hebei Dingzhou Power Generation Company Limited, Hebei 073099, China; xhitsumottk@gmail.com

**Keywords:** microwave resonant cavity, resonant frequency, pulverized coal mass concentration, TE_111_ mode

## Abstract

Pulverized coal mass concentration in the primary air pipe is one of the essential parameters for promoting furnace combustion efficiency. However, attaining accurate, real-time, and online detection for pulverized coal mass concentration remains challenging due to factors such as large pipe diameter and high flow rate. This study introduces a quasi-open microwave resonant cavity sensor. The principle and model were analyzed using the perturbation method, and the design and optimization were conducted with the simulation. A prototype and its test system were constructed, and the test results demonstrated good agreement between the simulations and experiments. The simulation revealed that the resonant frequency decreased monotonically from 861 to 644 MHz as mass concentration increased within 20%~80%, resulting in a change of about 3.62 MHz/1% under static mixture. The resonant frequency showed a drop from 21 MHz to 9 MHz with an increase in mass concentration under pulverized coal flow. Prediction models were developed and validated, showing the absolute values of the relative errors to be within 4% under operational scenarios. Additionally, the impact of the sensor on pulverized coal flow was evaluated, and it was found that the sensor structure had minimal impact on the flow in terms of velocity and the distribution of continuous flow. Finally, the long-term stability was assessed by examining the wear of the antennas and barriers. With inner barriers experiencing up to 2/3*d* wear, the resonant frequency drift ratio remained below 1.5%, corresponding to a mass concentration deviation of less than 3.2%.

## 1. Introduction

Excessive carbon dioxide emissions are widely recognized as a major contributor to global warming and climate change. In line with global environmental goals, China has set ambitious targets to reduce CO_2_ emissions by the year 2030 and achieve carbon neutrality by 2060 [1]. Despite these aspirations, China remains heavily reliant on coal and other fossil fuels as its primary energy sources, which places immense pressure on energy conservation efforts [2]. Thermal power plants, a cornerstone of China’s energy landscape, play a pivotal role in CO_2_ emissions due to their inherently energy-intensive nature. Hence, the carbon emissions stemming from the thermal power generation sector represent a substantial portion of the overall contributors to global warming [3]. In thermal power plants, pulverized coal is pneumatically conveyed through the primary air pipe within the boilers. Accurate assessments of coal concentration, velocity, distribution, and phase fraction within this primary air pipe are imperative for effective CO_2_ reduction strategies. In particular, achieving real-time and precise evaluations of pulverized coal mass concentration is pivotal for enhancing combustion stability and efficiency and addressing challenges related to combustion boiler corrosion and exhaust pollutants.

The mixture of pulverized coal particles and air can be considered as a gas-solid two-phase flow in the primary air pipeline of a plant. There are various methods for measuring the process parameters of gas–solid two-phase flow, including electrostatic and capacitance [4,5,6,7,8,9], optical [10,11], acoustic [12,13,14], and microwave techniques [15,16,17]. However, each of these methods has its limitations when applied in industrial environments. Electrostatic techniques, being passive in nature, are characterized by highly random signals. These signals are influenced by numerous factors, including primary air velocity, coal quality, particle fineness, humidity, primary air temperature, and pipe geometry. Under real-world operating conditions, the relatively high conductivity and velocity of coal particles exacerbate charge transfer and saturation effects, which significantly impact measurement accuracy. Capacitive techniques are prone to issues such as charge accumulation on coal particles, resulting in significant temperature-induced signal drift, which undermines measurement stability and accuracy. Optical techniques are highly susceptible to interference from solid particles such as pulverized coal dust, leading to challenges like light source contamination. Acoustic techniques, which rely on mechanical wave propagation, are strongly affected by the medium’s state, including temperature, particle fineness, velocity fluctuations, and local inhomogeneities. Among these techniques, microwave technology is considered one of the promising techniques for on-site applications due to robust anti-interference capability and high measurement accuracy [18]. Microwave technology detects the velocity, concentration, and mass flow rate information of gas–solid fluids by observing changes in signals such as amplitude, phase, and frequency resulting from the interaction between microwaves and the substance. This technique can be categorized into resonant and non-resonant approaches [19]. The resonant approach offers high sensitivity when measuring small changes in the dielectric constant of the medium. Considering the relatively modest variations in the dielectric constant of pulverized coal and air two-phase flows in primary air pipe, the latter was chosen for this study.

Researchers have conducted numerous studies on process fluid measurements utilizing the resonant method. Caroline Buschmuller et al. [20] introduced a microwave stray field sensor for pharmaceutical use in two different fluidized-bed dryers. The sensor employing microwave resonance technology enabled a continuous moisture measurement independent of the product density. John Austin et al. [21] proposed an open-structure reflection, resonance microwave sensor for monitoring both the moisture content and the effective bulk density of rapidly flowing microcrystalline cellulose with a high degree of accuracy. Johan Nohlert et al. [22] presented a microwave cavity resonator for the monitoring of the effective permittivity in closed metal vessels. Meanwhile, they proposed a microwave measurement system comprising a resonant cavity sensor operated in several high-order modes to detect objects in granular media flowing through pipes. A resonant probe, whose front features a resonant iris structure attached to the aperture, was utilized by Zubair Akhter et al. [23] to detect the concentration of solid contaminants in gas flows. In addition, Andreas Penirschke et al. [24,25] developed a novel mass-flow sensor based on a CRLH-TL-based metamaterial waveguide structure using TM_011_ resonant mode to accurately determine the mass-flow rate. Chao et al. [26] used a cylindrical resonant cavity operating in TM_110_ mode to detect the water cut of petroleum. Moreover, resonant technologies have many applications in other areas of flow measurement: for example, detection blood glucose concentration [27,28], water hardness [29], and salt and sugar in water [30]. Many sensor structures have been used in previous investigations, yielding positive outcomes. Unfortunately, however, the microwave resonant method still has very few applications in cases involving large pipe diameters and high flow velocity in the primary air of thermal power plants.

This study aims to develop a quasi-open microwave cavity sensor to measure the pulverized coal mass concentration in primary air scenarios. First, the measurement principle and theoretical model are discussed. A 3D sensor model is then established and optimized using the finite element method. Following this, the sensor is fabricated and the test system is constructed. To determine measurement properties, extensive simulations are conducted under static mixture and pulverized coal flow conditions. Prediction models are developed, and their associated errors are estimated. Additionally, the sensor performance, particularly its invasiveness, is explored. Finally, different levels of wear on the sensor rods are assessed to evaluate the stability of measurements over time.

## 2. Measurement Principle and Model

The resonant frequency and quality factor are crucial parameters of a resonant cavity. The dielectric constant can be determined by these two parameters: The real part is determined by the resonant frequency, and the imaginary part is determined by the quality factor [31]. The perturbation method, which includes perturbations in resonant frequency and quality factor, is commonly used to calculate the dielectric constant in the resonant cavity. However, the quasi-open cavity design inherently results in a relatively low Q-factor, which limits the sensor’s effective sensing range. In contrast, resonant frequency is easier to measure with higher precision and a broader dynamic range. Therefore, this study adopts the perturbation method focusing on resonant frequency. The primary air pipe, made of metal, serves as the circumferential wall of the resonant cavity, while the embedded outer barriers within the pipe act as the axial boundaries. This arrangement makes the material perturbation method more suitable for measurement than those perturbed by shape or volume due to the fixed dimensions of the cavity. According to the material perturbation method, the dielectric constant of materials can be estimated by the resonant frequency. Particularly, when the material is a multiphase mixed medium, its dielectric constant can be obtained, and then the mass fraction of one phase can be calculated using the equation of effective dielectric constant.

**Figure 1:**
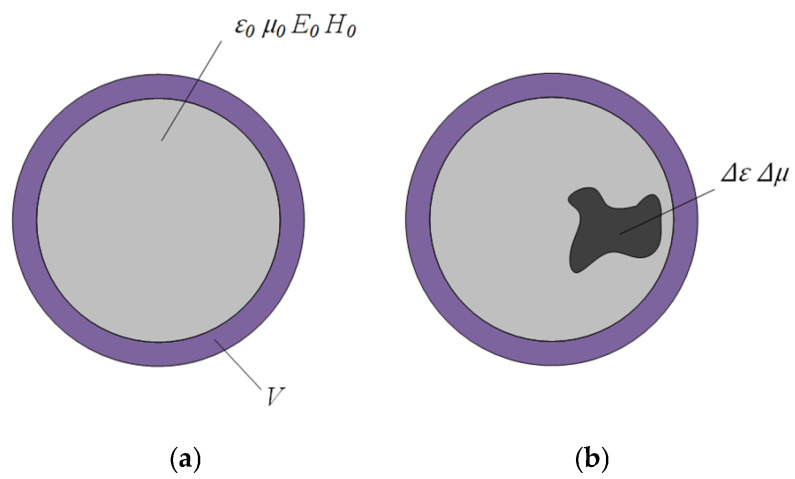
Coal particle flow perturbation on the cross-section of the resonant cavity: (**a**) Particles flow unperturbed; (**b**) particles flow perturbed.

Figure 1 shows slight variations in dielectric constant or permeability (∆*ε* or ∆*μ*) owing to the changes of mass concentration *m_c_*. In the figure, purple denotes the circumferential wall of the cavity, light grey denotes initial coal particle flow, and dark grey signifies perturbed coal particle flow. *V* is the volume of the cylindrical load in the pipe. $\bar{E_{0}}$ and $\bar{H_{0}}$ are the electric and magnetic fields of the unperturbed cavity, while *μ_0_* and *ε_0_* are the permeability and dielectric constants of the medium before perturbation, respectively.

The resonant frequency changes due to perturbation, which can be calculated using Maxwell’s curl equations [32]. It can be expressed as follows:

(1) $$\frac{f_{r}-f_{r0}}{f_{r}}=\frac{-\int_{V}\left(\Delta \varepsilon \bar{E}\cdot \bar{E_{0}}+\Delta \mu \bar{H}\cdot \bar{H_{0}}\right)dv}{\int_{V}\left(\varepsilon_{0}\bar{E}\cdot \bar{E_{0}}+\mu_{0}\bar{H}\cdot \bar{H_{0}}\right)dv}$$

where *f_r_*_0_ and *f_r_* are the resonant frequency before and after perturbation, respectively. $\bar{E}$ and $\bar{H}$ are the electric field and magnetic field of the perturbed cavity. If ∆*ε* and ∆*μ* are exceedingly small, the electric field and magnetic field perturbed can be replaced by the original field, $\bar{E}$ ≈ $\bar{E_{0}}$ and $\bar{H}$ ≈ $\bar{H_{0}}$. In addition, the relative magnetic permeability of coal particles is 1, which is the same as air; therefore, ∆*μ* = 0. This allows for further simplification as follows:

(2) $$\frac{f_{r}-f_{r0}}{f_{r}}\approx \frac{-\int_{V}\left(\Delta \varepsilon |\bar{E_{0}}{|}^{2}\right)dv}{\int_{V}\left(\varepsilon_{0}|\bar{E_{0}}{|}^{2}+\mu_{0}|\bar{H_{0}}{|}^{2}\right)dv}$$

The term *C* is introduced, thus

(3) $$C=\frac{\int_{V}{\left|E_{0}\right|}^{2}dv}{\int_{V}\left(\varepsilon_{0}|\bar{E_{0}}{|}^{2}+\mu_{0}|\bar{H_{0}}{|}^{2}\right)dv}$$

And Δ*ε* can be replaced by the deviation between the dielectric constant of a mixture containing pulverized coal and air (*ε_mix_*) and the dielectric constant *ε*_0_ in the original field. Therefore, Equation (2) can be simplified as:

(4) $$\varepsilon_{mix}=\varepsilon_{0}-\frac{1}{C}\left(\frac{f_{r}-f_{r0}}{f_{r}}\right)$$

It is observed that an increase in the dielectric constant *ε_mix_* results in a decrease in the resonant frequency, as derived from Equation (4). If the resonant frequency *f_r_*_0_ and *f_r_* are measured, then *ε_mix_* can be obtained.

In order to develop a prediction model for mass concentration *m_c_*, an equation for effective dielectric constant is necessary. *ε_mix_* is related to the proportion of air and pulverized coal, their dielectric constants, and their distribution. Coal is pulverized into powder by the coal mill. Although the powder particles exhibit a random shape, they typically have a very small particle size (<100 um). Consequently, the coal powder (pulverized coal) can be regarded as being homogeneously mixed with the continuous air medium in a specific spatial domain. Given that the derivation is independent of particle shape, the Looyenga formula [33] is universally applicable and particularly suited for homogeneous mixtures. Hence, *ε_mix_* can be estimated by substituting the dielectric constants of pulverized coal (*ε_c_*) and air (*ε_a_*) as follows:

(5) $$\varepsilon_{mix}={\left[(\varepsilon_{c}^{1/3}-\varepsilon_{a}^{1/3})m_{c}+\varepsilon_{a}^{1/3}\right]}^{3}$$

The relative dielectric constant of air *ε_a_* is about 1, while that of the pulverized coal *ε_c_* is approximately 2.7 [34]. Equation (6) can be derived further:

(6) $$m_{c}=\frac{\varepsilon_{mix}^{\frac{1}{3}}-1}{2.7^{\frac{1}{3}}-1}$$

**Figure 2:**
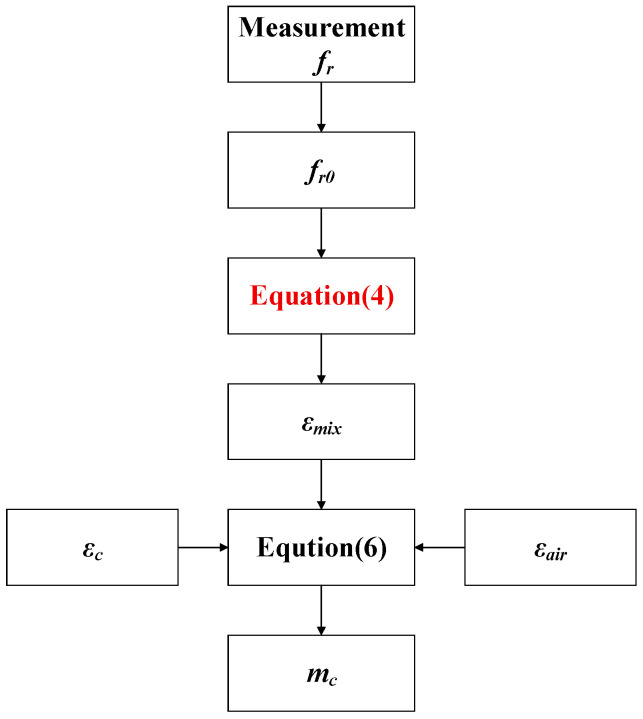
Calculation procedure for pulverized coal mass concentration.

Provided that the resonant frequency is measured from the sensor, *m_c_* can be determined using Equations (4) and (6). The numerical procedure adopted in this study is depicted in Figure 2.

## 3. Sensor Design and Optimization

### 3.1. Sensor Structure and Operating Mode

**Figure 3:**
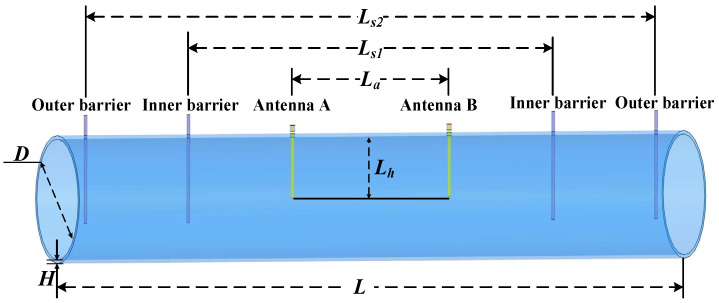
Sensor structure of the quasi-open resonant cavity.

Figure 3 illustrates the sensor structure that is designed. A cylindrical pipe with a diameter of *D* = 207 mm and a thickness *H* = 6 mm is chosen as the wall of the sensor. A pair of monopole antennas (A and B), based on the one-quarter operating wavelength, are chosen as the transmitter and receiver. These antennas are inserted into the pipe, with distance and depth denoted as *L_a_* and *L_h_*. The antennas are constructed of copper, and their encasing material is polytetrafluoroethylene. In addition to antennas A and B, there are two inner copper rods and two outer copper rods (named inner barriers and outer barriers) positioned, with distances denoted as *L_s_*_1_ and *L_s_*_2_, respectively. The arrangement serves for electrical short-circuiting and limits the scattering of electromagnetic fields from the two ends of the resonant cavity.

**Figure 4:**
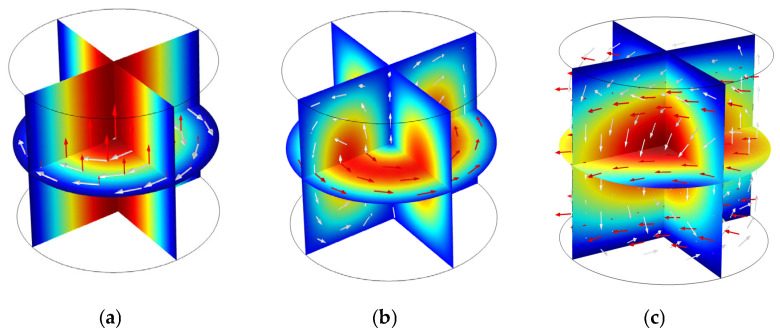
The electromagnetic field distribution of resonant modes: (**a**) TM_010_; (**b**) TE_011_; and (**c**) TE_111_. (The red arrows represent the electric field line, while the white arrows represent the magnetic field line.).

The resonant mode, which represents a spatial distribution of electromagnetic fields in the defined cavity, needs to be determined for sensing the mixture media. Three modes, namely TM_010_, TE_011_, and TE_111_, are frequently used, where TE stands for transverse electric wave and TM stands for transverse magnetic wave. Figure 4 illustrates the electric field and magnetic field distribution for the three modes. While the TM_010_ mode theoretically offers the highest sensitivity, its axial symmetry and significant energy leakage under quasi-open boundary conditions greatly hinder its practical applicability. The TE_011_ mode, although more stable, exhibits insufficient electric field coupling with centrally located flow media. In contrast, the TE_111_ mode presents slightly lower sensitivity than TM_010_ and a lower quality factor than TE_011_, but its transverse electric field distribution is more compatible with the quasi-open cavity structure. Moreover, TE_111_ demonstrates greater modal stability and is less susceptible to interference from competing modes compared to TE_011_. The comparative analysis supports the selection of TE_111_ mode as the optimal trade-off among sensitivity, structural adaptability, and mode separation. In addition, the mode can quickly be excited by the antenna, provided that the tip of the antenna is placed at the position of maximum electric field intensity. It is important to note that the resonant frequency of TE_111_ is directly related to the diameter of the pipe and the length of the cavity. Therefore, for sensors of different sizes, optimal performance requires adjustments in sensor parameters and design.

### 3.2. Optimization of Sensor Structural Parameters

**Figure 5:**
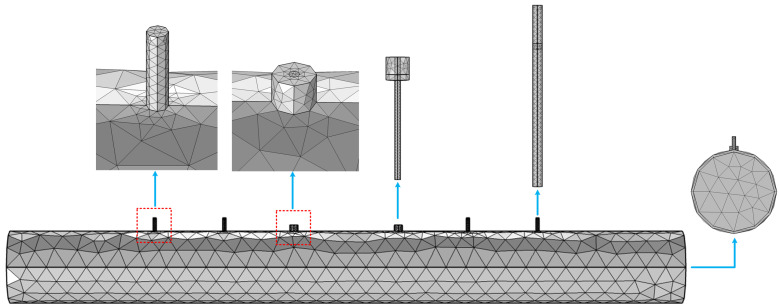
3D FEM modeling and meshing of the sensor.

**Figure 6:**
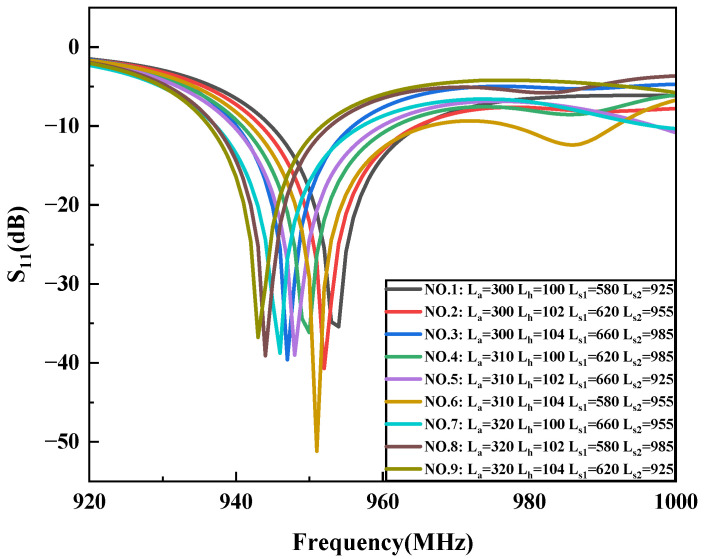
Simulation results of nine sensor structures from the orthogonal array.

**Figure 7:**
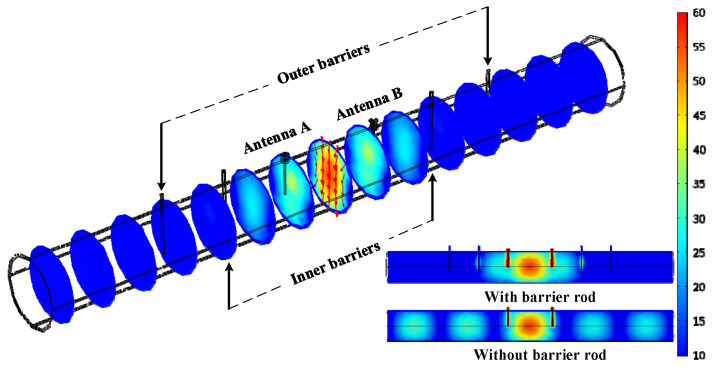
The distribution of the electric field in the resonant cavity.

As for the resonant cavity sensor, the diameter is known, but the location information about the antennas and barriers is still unknown. Thus, the key parameters, including the distance (*L_a_*) between the two antennas and the distances (*L_s_*_1_ and *L_s_*_2_) between the inner and outer barriers, will be optimized and determined. Furthermore, the insertion depth (*L_h_*) in the pipeline also needs to be determined. COMSOL (Version 5.6) Multiphysics^®^ simulation software using the finite element method, is employed to design and optimize the geometric structure of the sensor. The model is built and meshed with 36,551 elements, as shown in Figure 5. To prevent interference from the adjacent mode TM_010_ and TE_112_, *L_a_* and *L_h_* were initially designed as 1.5*D* and 0.5D, respectively. Consequently, a wavelength of 307 mm is obtained. To ensure optimal short-circuit conditions, the barriers were positioned at integer multiples of the half-wavelength. *L_s_*_1_ and *L_s_*_2_ were initially set as 1.5*D + λ* and 1.5*D +* 2*λ*. The orthogonal experimental method was employed to optimize the sensor structure, with each experimental factor set at three levels according to the initial configuration, as detailed in Table 1. An L9 (3^4^) orthogonal array was adopted, resulting in nine groups of sensor configurations with different factor combinations. The S_11_ parameter was selected as the evaluation index. The simulation results are presented in Figure 6. It can be observed that the S_11_ parameter reaches its lowest value of −51.18 dB in Test No. 6. Therefore, considering the interaction of all factors, the optimal combination of structural parameters is determined to be *L_s_*_1_ = 580 mm, *L_s_*_2_ = 955 mm, *L_a_* = 310 mm, and *L_h_* = 104 mm. Figure 7 illustrates the electric field distributions after optimation. Obviously, the electric field of the TE_111_ mode is primarily concentrated within the two antennas, and the inner barriers effectively create a short circuit at both ends. The inclusion of the outer barriers further ensures that outside electromagnetic wave propagation is minimal. In contrast, without barrier rods, it can be observed that the electromagnetic field is transmitted out in the TE_111_ mode.

## 4. Experimental Test

### 4.1. Sensor and Test System Construction and Experiment Setup

**Figure 8:**
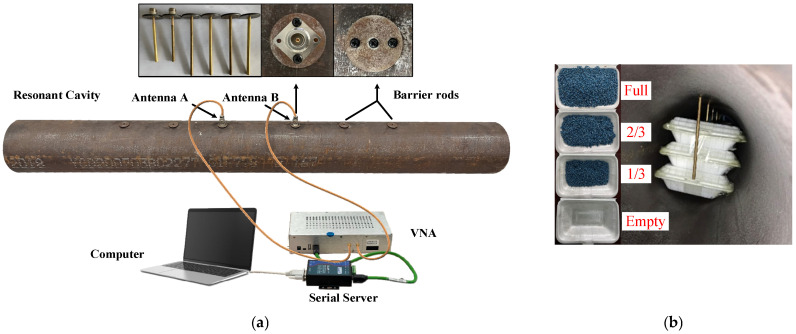
Sensor test system and experiment setup: the (**a**) sensor and its test system and (**b**) experiment setup.

The sensor was fabricated, as shown in Figure 8a. It consists of a pipe, antennas, and barrier rods. The pipe is constructed from carbon steel. The antenna is formed by a copper rod, circular carbon steel gaskets, and coaxial connectors. The barriers are constructed from copper rods, with one end drilled to accommodate an internal thread for attachment to a bolt and fitted with circular carbon steel gaskets. The diameter of both the antenna and barrier rods is 6 mm. Additionally, the test system comprises a computer, Vector Network Analyzer (VNA)—DEVISER E7200A (measuring range: 300 KHz to 3 GHz, frequency resolution: 1 KHz), serial server, and sensor, coordinated to form the test system. Controlled by a computer, the VNA launches microwave signals and receives signals containing coal concentration information. Lastly, S-parameter data are recorded. The repeatability tests are conducted to check the measurement stability of the sensor and test system. In the empty pipe, 10 sets of data are collected and tested per day for three consecutive days. The repeatability calculated from 30 datasets is less than 0.072%.

**Figure 9:**
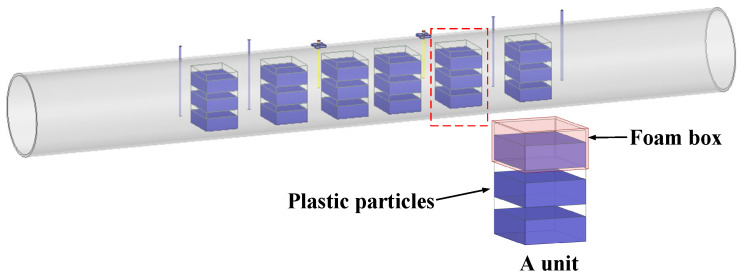
3D FEM model of the sensor with TE_111_ mode distribution.

It is challenging to maintain an even distribution of pulverized coal under static tests. To address this, a compromise approach was employed. The test medium is simulated by a foam box filled with plastic pellets. The foam has a dielectric constant close to 1, similar to air, while the plastic particles have a dielectric constant of 2.4 and a diameter of approximately 2 mm. Some units, each of which comprises three foam boxes, are positioned inside the pipe, as illustrated in Figure 8b. The volume ratios of 0, 1/3, 2/3, and 1 are arranged inside the box to test the sensor response. For follow-up studies, the simulation is conducted simultaneously; the model is shown in Figure 9.

### 4.2. A Comparison Between Experiments and Simulations

**Figure 10:**
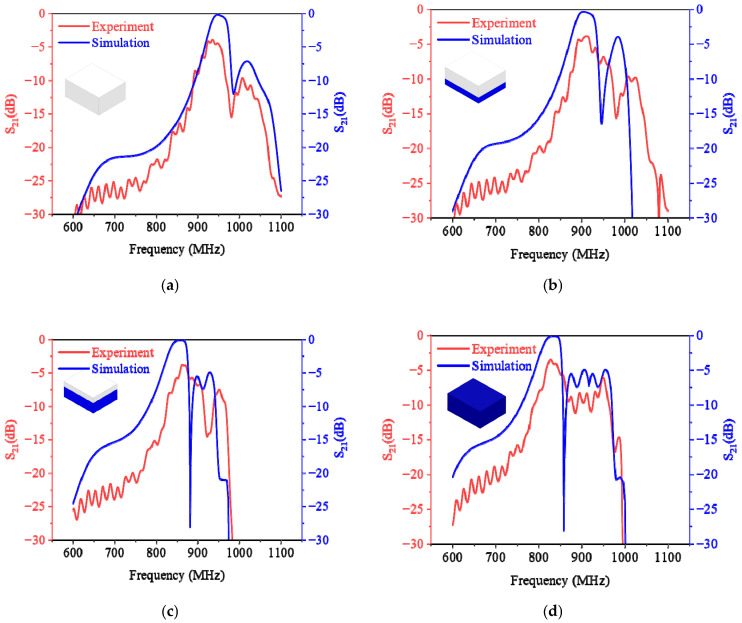
Experimental and simulated S_21_ curves in four filling ratios: (**a**) empty box; (**b**) 1/3 box; (**c**) 2/3 box; and (**d**) full box.

**Figure 11:**
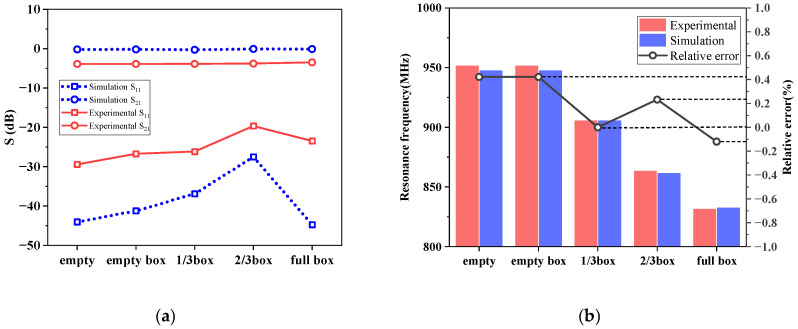
Comparison of S parameter and resonant frequency (experiment and simulation): (**a**) comparisons of S parameters; (**b**) comparisons of resonant frequencies.

To conduct more extensive and in-depth research, the simulation method must be used. Regarding this, the consistency between the simulation and experiment will be examined carefully. Figure 10 illustrates the S_21_ (forward transmission coefficient) curves derived from both simulation and experimental data across four volume ratios. It can be observed that these curves share nearly identical trends and shapes, but there is a slight discrepancy, especially in the experimental curves, which manifest many high-harmonic contents and a lower peak. This divergence can be attributed to the roughness on the inner surface of the pipeline, contributing to both asymmetrical reflection and signal loss. Furthermore, Figure 11 presents a comparison of S_11_, S_21_, and resonant frequencies obtained from both simulations and experiments. As depicted in Figure 11a, the S_11_ and S_21_ trends align with the increasing mass concentration (*m_c_*). The discrepancy remains. The differences in S_11_ range from 7.85 dB to 21.26 dB, and those in S_21_ range from 3.37 dB to 3.72 dB. From Figure 11b, it is evident that the resonant frequency monotonically decreases with the increase in plastic pellets in the foam box. The variation in resonant frequencies between the simulation and experiment ranges only from 0.12% to 0.42%. This negligible difference can be attributed to the careful adjustment of the simulation model’s size to closely resemble the practical experiment’s. Therefore, the feasibility of utilizing the simulation method to investigate the sensor is affirmed, given that the resonant frequency, as the main parameter, exhibits the most significant deviation of less than 0.42%. Building on this, the COMSOL simulations will be performed to investigate the measurement properties of the sensor.

## 5. Result and Analysis

### 5.1. Measurement Property for Static Mixtures

In the pneumatic conveying process of a thermal power plant, raw coal is pulverized in the coal mill and then transported to the furnace by air, which acts as the carrier, usually at a temperature of approximately 80 °C. Throughout this process, the density of coal particles ranges from 1400 kg/m^3^ to 1600 kg/m^3^. The diameter of pulverized coal particles is around 10 μm to 200 μm. The concentration inside the pipeline falls within the middle to low range, generally categorized as dilute-phase pneumatic conveying. The velocity typically ranges from about 20 to 30 m/s. Thermal power plants commonly stipulate that the moisture content of coal should be within 8%. After grinding and heating by the primary air, the moisture content of pulverized coal in the pipeline is very low, resulting in minimal agglomeration of coal particles. Additionally, regarding coal powder transportation, power plants have well-defined parameters such as particle material, size, shape, humidity, conveying distance, pipeline material, and pipeline layout. These factors yield relatively favorable conditions for conducting measurements. As a result, a straight pipe section can easily be chosen, allowing for the well-dispersed and uniform development of coal powder in the pipeline for measurement. For quantitative analysis, the mass concentration of pulverized coal is set between 20% and 80% under extreme conditions, with the influence of coal powder moisture content on the measurement being disregarded.

**Figure 12:**
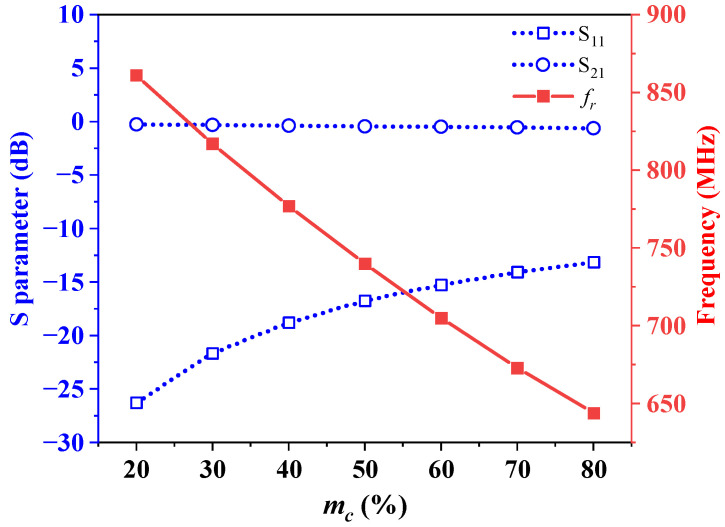
S parameter and resonant frequency at various mass concentrations.

The static mixture (SM) simulations, which treat the mixture of primary air and pulverized coal as a distinct substance, were conducted to evaluate the measuring property of the sensor. The dielectric constant of the substance, *ε_mix_*, needs to be known in advance. Equation (5) can be used to calculate *ε_mix_* with *m_c_* incrementing in steps of 10% from 20% to 80%. The calculated and simulated results are presented in Table 2, while Figure 12 illustrates the resonant frequencies and the S_11_ and S_21_ parameters. The findings illustrate that a clear pattern emerges: As the mass concentration (*m_c_*) increases from 20% to 80%, the dielectric constant of the static mixtures steadily rises from 1.2527 to 2.2584, accompanied by a consistent decrease in resonant frequency from 861 to 644 MHz. Simultaneously, the S_11_ parameter exhibits a continuous increase from −26.269 dB to −13.123 dB, while the S_21_ parameter decreases monotonically from −0.26 to −0.92 dB. Within the operation range, the S_11_ parameter is far less than −10 dB and the S_21_ parameter is more than −1 dB, achieving the expected resonance state. A change of 1% in mass concentration corresponds to an average shift in resonant frequency of 3.62 MHz. These observations highlight the measurable nature of the quasi-open resonant cavity sensor, showcasing its high sensitivity in detecting the mass concentration of pulverized coal.

### 5.2. Measurement Property for Pulverized Coal Flow

**Figure 13:**
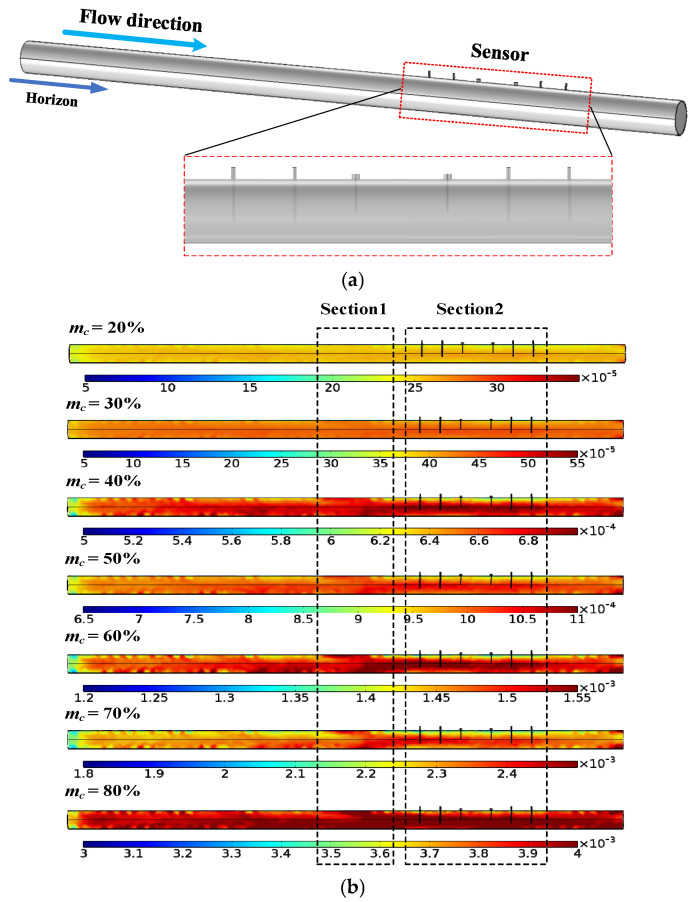
Simulations with pulverized coal flow: (**a**) 3D FEM model for simulations; (**b**) pulverized coal distributions in the pipeline.

To investigate the sensor response in the context of pulverized coal flow (PCF), simulations coupling flow field and electromagnetic field are employed. A sufficiently long pipe (5 m) is placed in front of the sensor to ensure a fully developed flow; the 3D model is depicted in Figure 13a. The Euler–Euler approach, which involves RANS,k-ε is chosen. The continuous phase is set as air, while the dispersive phase is pulverized coal with a density of 1400 kg/m^3^ and a diameter of 90 μm. Both phases exhibit a velocity of 30 m/s. The boundary condition is set as “pressure with no viscous stress,” and a no-slip condition is established between the continuous flow and the wall. Gravity and drag force are considered in the simulation due to their practical significance. A user-controlled mesh is defined, resulting in a total of 44,253 elements.

Figure 13b illustrates the distribution of pulverized coal in the pipeline across a mass concentration (*m_c_*) range from 20% to 80%. The color scale represents the volume percentage converted by mass concentration. As the flow progresses from the straight pipe section to Section 1, reaching full development, the distributions appear similar. However, upon advancing to Section 2, differences become apparent. In this section, the upper part of the pulverized coal distribution becomes sparse, while the lower part becomes dense on the axial plane where the sensor is located. This is due to the obstruction caused by the antennas and barriers. The effect is especially pronounced for *m_c_* = 40%, 50%, 60%, and 70%, while it is minimal for *m_c_* = 20%, 30%, and 80%. Nevertheless, regardless of the *m_c_* value, once the flow passes through the sensor section, the flow distribution immediately returns to normal. This analysis highlights the distinct distribution patterns of pulverized coal flow compared to static mixtures.

**Figure 14:**
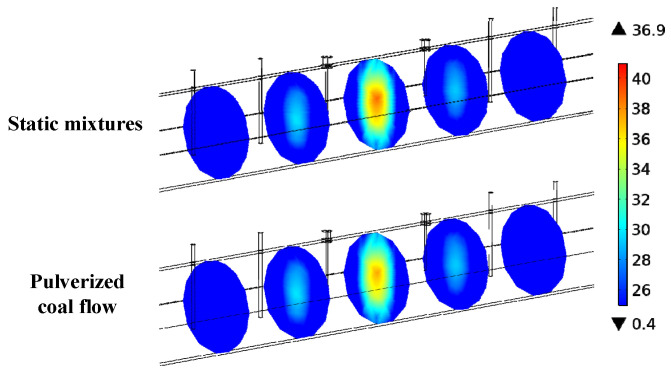
Electronic fields under SM and PCF simulations (*m_c_* = 0.3).

To examine the sensor’s responses to pulverized coal flow, a coupled simulation between flow and electromagnetic fields is conducted. Figure 14 displays the electric field distribution of *m_c_* = 30% in static mixture (SM) simulation and pulverized coal flow (PCF) simulation. The figure shows that, in both situations, the resonant mode TE_111_ in the resonant section remains consistent. This forms the basis for the applicability of this technique to real fluid conditions. However, in PCF simulations, the central electric field intensity weakens. This may be because the uneven spatial distribution disperses the concentration of the electric field intensity. As a result, there are differences in resonant frequencies between SM and PCF. As shown in Table 3, the difference decreases from 21 MHz to 9 MHz as *m_c_* increases from 20% to 80%. The average frequency difference is around 14 MHz. This phenomenon may be attributed to blockage from antennas, resulting in a reduction in the distribution of pulverized coal in the axial plane where the antenna is located (i.e., the sensor’s most sensitive area), and this effect gradually diminishes with an increase in *m_c_*.

### 5.3. Prediction Models

The close agreement between the simulation and experimental results underscores the reliability of acquiring sensor test data through simulations. Consequently, this data can be utilized in developing predictive models for mass concentration. From Equation (4), a linear relationship exists between $\frac{f_{r}-f_{r0}}{f_{r}}$ and *ε_mix_*. For improved accuracy, Equation (4) is reformulated as follows:

(7) $$\frac{f_{r}-f_{r0}}{f_{r}}=a{\varepsilon_{mix}}^{2}+b\varepsilon_{mix}+c$$

where a, b, and c are the underdetermined coefficients. *f_r_*_0_ = 951 MHz at *m_c_* = 0. The linear fitting method can be used to accurately obtain the prediction models.

For the SM situation, 61 data points of the dielectric constant’s dependence on resonant frequency are obtained with a step of 1% from *m_c_* = 20% to *m_c_* = 80%. Every third point is extracted, resulting in a total of 21 points for the training dataset, while the remaining points form the validation dataset. Consequently, a = −0.3768 and b = 0.3512 are obtained, with a correlation coefficient R^2^ = 0.9999. The prediction model is as follows:

(8) $$\frac{f_{r}-f_{r0}}{f_{r}}=0.0528{\varepsilon_{mix}}^{2}-0.561\varepsilon_{mix}+0.5071$$

For the PCF situation, 13 data points of the dielectric constant’s dependence on resonant frequency are generated, with a step of 5% from *m_c_* = 20% to *m_c_* = 80%. One point is extracted after every data point, and a total of seven points are collected into the training dataset, while the remaining points constitute the validation dataset. According to the characteristics of the curve, the natural logarithm (ln) function was introduced. The resulting prediction model is shown in Equation (9), yielding a correlation coefficient of R^2^ = 0.9977.

(9) $$\frac{f_{r}-f_{r0}}{f_{r}}=-0.617\ln(\varepsilon_{mix})+0.0154$$

**Figure 15:**
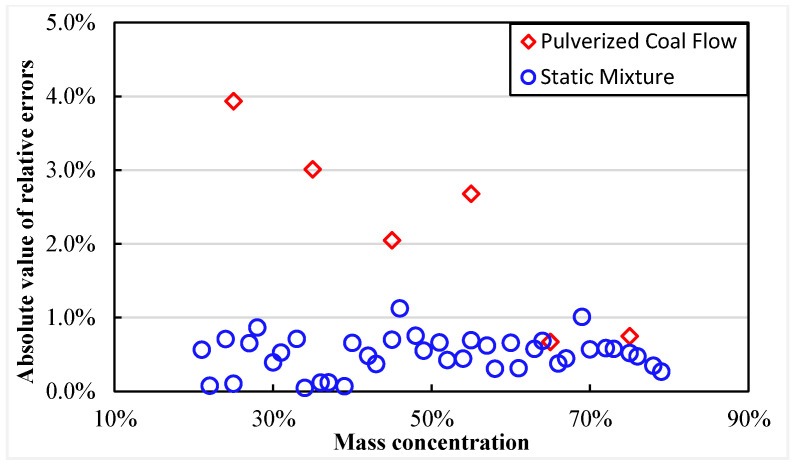
The mass concentration prediction errors under SM and PCF conditions.

Figure 15 depicts the absolute values of relative errors predicted by Equations (8) and (9) using the validation dataset for both SM and PCF conditions, respectively. The figure shows that, in both situations, the absolute values of the relative errors in predicting mass concentration are within 4%. This signifies that the absolute errors for all predicted points are confined within ±1.2%. More specifically, relative errors less than ±1.2% under SM conditions show the superior performance. These findings suggest that the developed models exhibit a wide range of predictive capacities, alongside precise prediction abilities for common conditions.

### 5.4. The Impact of the Sensor on Pulverized Coal Flow

**Figure 16:**
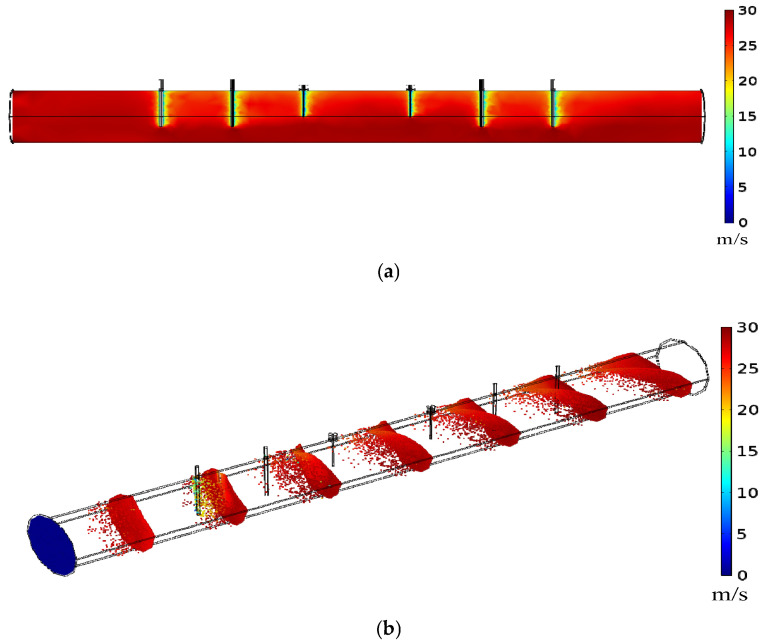
Particle flow velocity distribution using the Lagrangian approach: (**a**) air velocity distribution; (**b**) particle velocity distribution.

The effect of the quasi-open sensor on the flow of pulverized coal needs to be examined carefully. As described in Section 5.2, the Euler–Euler approach simulation was used to obtain pulverized coal flow distribution. The influence exerted by the sensor is concentrated on the section of the sensor within a partial range of conditions 40%~70%. Following that, the Lagrangian approach, which follows individual particles as they move, was used to examine details regarding the impact. The particle tracing module, which involved “turbulent flow k-ε” and “particle tracking for fluid flow,” was employed in simulation experiments. The left and right side of the pipe (2 m) were set as the “inlet and outlet” boundaries, respectively. The air velocity was set to 30 m/s at the inlet, and the maximum number of particles was 10,000. The pressure at the outlet was barometric pressure. The wall condition was selected as no-slip. When pulverized coal particles enter the pipeline, the drag force and gravity are the primary influencing factors. Finally, “fluid–particle interaction” is added to the “multi-physics field”. The results of the simulation experiment are shown in Figure 16.

Figure 16a shows the distribution of the air flow velocity. When the air encounters antennas and barriers, its velocity drops from 30 m/s to about 15 m/s, and then quickly returns to its original speed after only a short distance. Figure 16b shows the trajectory of the particles and their velocity profiles. When entering the pipeline, coal particles undergo acceleration due to the drag force exerted by the air. Most particles maintain the same velocity and form a particle surface, while a few particles lag. As the coal particles pass through the first inserted barrier rod, a few particles experience an obvious reduction in velocity, especially the particles in the barrier plane, while the majority maintain a forward flow of approximately 30 m/s. After the following several barriers and antennas, the spatial location of particles shows significant divergence in their forward movement. This results in the particle distribution stretching axially and the particle surface bending due to obstacles. However, the particle velocity promptly returns to approximately 30 m/s, sustaining onward movement upon traversing this sensing section. This indicates that the velocity is minimally affected by the pulverized coal flow. Considering the continuous replenishment of particles in actual flow, resembling the Euler–Euler approach where particles move continuously and reach full development, the impact of the sensor on pulverized coal flow is swiftly rectified. This phenomenon occurs because the cross-sectional area available for particle passage significantly surpasses the obstructed plane blocked by barriers. As a result, it can be inferred that the quasi-open resonant cavity structure designed has minimal perturbation on the transport of coal particles.

### 5.5. Long-Term Stability of the Sensor

**Figure 17:**
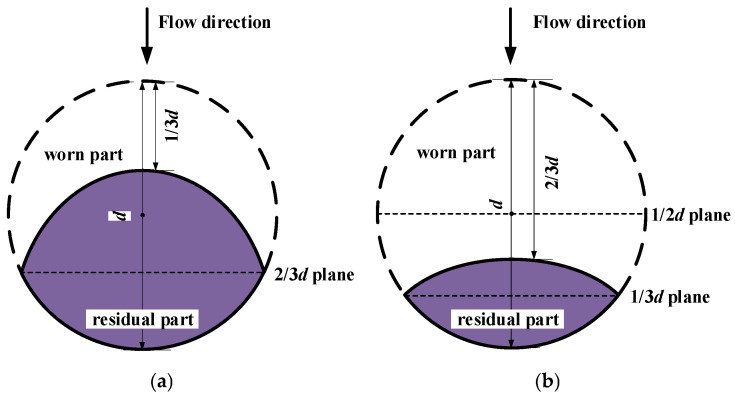
Outline of the worn rod: (**a**) 1/3*d* wear (ellipticity = 0.25); (**b**) 2/3*d* wear (ellipticity = 0.33).

Although the cross-sectional area of the antennas and barriers within the quasi-open microwave cavity is minimal compared to that of the pipeline, wear on the sensor’s antennas and barriers is inevitable due to the long-term impact of pulverized coal during operation. Wear is a major issue affecting the stability of the sensor over time. Thus, the long-term stability of the sensor can be assessed by examining changes in resonant frequency under varying levels of wear. The barriers are numbered No. 1 and No. 2, starting from one end of the pipe inward. Figure 17 shows the dimensions and shapes of the worn rods. Antennas and barriers are worn by 1/3*d* or 2/3*d* of their original diameters, forming an elliptical arc shape on the windward side. According to the wear behavior, their ellipticities are set as 0.25 and 0.33, respectively. When the structure is unworn, the electromagnetic effective boundary location (EBL) plane corresponds to the 1/2*d* plane. For the case of 1/3*d* wear, the residual part is a local ellipse with a major axis of 4/3*d* and a minor axis of *d*; in this scenario, the EBL plane shifts to the 2/3*d* plane. When the wear reaches 2/3*d*, the residual part is the local ellipse with a major axis of *d* and a minor axis of 2/3*d*, and the EBL plane shifts to the 1/3*d* plane. In the simulation experiment, 10 cases of worn rods are simulated within an empty cavity, with the cases and results presented in Table 4.

Table 4 shows that the resonant frequency decreases from 951 to 937 MHz as wear increases from 1/3*d* to 2/3*d* of all rods in Cases 1–2, with the resonant frequency drift ratio remaining below 1.5% (corresponding to a mass concentration deviation of less than 3.2%). In Cases 3–5, 1/3*d* wear on No. 1 and No. 2, as well as 2/3*d* wear on No. 1, have no effect on the resonant frequency. However, 2/3*d* wear on No. 2 results in a noticeable increase in the resonant frequency in Case 6. Despite this, the resonant frequency drift ratio remains below 1.5% across Cases 1–6. When No. 1 and No. 2 (the barriers at one end) are worn from 1/3*d* to 2/3*d* in Cases 7–8, the resonant frequency begins to increase, with a drift ratio of 1.05%. In Cases 9–10, cross-wearing of No. 1 and No. 2 by 1/3*d* and 2/3*d,* respectively, results in changes to the resonant frequency. This variation can be clearly observed from the EBL plane. In all cases where wear results in a drift ratio in the resonant frequency, the EBL plane of No. 2 corresponds to the 1/3*d* plane. When No. 1 or all antennas and barriers are located on the 1/3*d* plane, the drift ratio becomes more pronounced. These observations indicate that wear of the inner barriers leads to more significant changes in the resonant frequency. The analysis further suggests that the inner barriers serve as the actual electromagnetic boundary of the resonant cavity. When wear on the inner barriers exceeds 2/3*d* on the EBL 1/3*d* plane facing the flow direction, the effective cavity axial length is reduced, causing an increase in the resonant frequency. Therefore, it can be concluded that most wear on the antennas and outer barriers has minimal impact on the sensor’s resonant frequency response, thereby ensuring its long-term operational stability. However, when wear of the inner barriers exceeds 2/3*d* (1/3*d* plane), closer monitoring of the sensor’s condition is recommended to maintain accuracy and reliability.

## 6. Conclusions

This paper investigated a novel quasi-open resonant cavity designed for measuring the mass concentration of pulverized coal. The principle and model development, sensor design, experimental testing, and analysis using simulations were performed. The main findings are summarized as follows: (1) A novel quasi-open resonant cavity sensor was developed. The sensor, operating in TE_111_ mode, consists of barriers, antennas, and the pipeline. Under static mixture conditions, the resonant frequency consistently decreases from 861 to 644 MHz as the mass concentration increases from 20% to 80%, with an average shift of 3.62 MHz/1%. Under pulverized coal flow, the measured resonant frequency drops from 21 MHz to 9 MHz as *m_c_* increases, compared to static mixtures. (2) The prediction models for the sensor detecting the mass concentration were proposed. Models for both static mixture and pulverized coal flow situations were built based on the simulation data. The validated results show that the absolute values of the relative errors in predicting mass concentration are within 4%. (3) The impact of the sensor on pulverized coal flow was evaluated using both Euler–Euler and Lagrangian method simulations. The results revealed that the sensor structure has an impact on the velocity and distribution of continuous flow within the plane of barriers in the 40%~70% range, but the impact is swiftly rectified. It can be concluded that the designed quasi-open resonant cavity structure has minimal perturbation on the transport of coal particles. (4) The long-term stability was evaluated by examining different levels of wear on the antennas and barriers. The findings suggest that when all rods experience up to 2/3*d* wear, the resonant frequency drift ratio remains below 1.5% (corresponding to a mass concentration deviation of less than 3.2%). The wear of the inner barriers is a major factor, as they form the primary boundary of the cavity. Therefore, special attention should be shown to the wear of inner barriers when it exceeds 2/3*d*.

This study presents a new measuring method for the primary air flow detection technology used in power plants, which has the potential to advance the development of real-time, precise, and dynamic detection systems for thermal power plants. However, potential challenges, particularly those related to factors such as humidity, temperature, and coal rank, need to be further studied and addressed under actual operating conditions. Additionally, more rigorous and comprehensive experimental validation studies of the simulations under real flow conditions are urgently needed. Future work will focus on conducting tests in real power plant environments to thoroughly evaluate these challenges and issues. Despite these limitations, this research is expected to offer valuable insights and contribute meaningfully to the field.

## Figures and Tables

**Table 1:** Preset values and sweep values for each parameter.

Parameters	Preset Values	Scann Range	Scan Step
*L_a_*	1.5D	290–350 mm	10 mm
*L_h_*	0.5D	94–114 mm	2 mm
*L_s_*_1_	1.5D + *λ*	550–650 mm	10 mm
*L_s_*_2_	1.5D + 2*λ*	875–975 mm	20 mm

**Table 2:** Datasheet of resonant frequency and S parameter.

*m_c_* (%)	*ε_mix_*	*f_r_* (MHz)	S_11_ (dB)	S_21_ (dB)
20%	1.2527	861	−26.269	−0.26278
30%	1.3937	817	−21.658	−0.30548
40%	1.5448	777	−18.778	−0.37282
50%	1.7065	740	−16.737	−0.44362
60%	1.8791	705	−15.252	−0.47397
70%	2.0629	673	−14.051	−0.52664
80%	2.2584	644	−13.123	−0.61999

**Table 3:** Resonant frequency differences between SM and PCF simulations.

*m_c_* (%)	SM (MHz)	PCF (MHz)	Differences (MHz)
20	861	840	21
30	817	798	19
40	777	762	15
50	740	727	13
60	705	693	12
70	673	663	10
80	644	635	9

**Table 4:** Datasheet on the wear of antennas and barriers.

Case	Condition	Resonant Frequency (MHz)	Resonant Frequency Drift Ratio	No. 1 EBL	No. 2 EBL	S_11_
Case 0	Unworn	951	0.00%	1/2*d* plane	1/2*d* plane	−51.1785
Case 1	All 1/3*d*	951	0.00%	2/3*d* plane	2/3*d* plane	−40.2692
Case 2	All 2/3*d*	937	−1.47%	1/3*d* plane	1/3*d* plane	−36.8934
Case 3	No. 1: 1/3*d*	951	0.00%	2/3*d* plane	1/2*d* plane	−48.2049
Case 4	No. 1: 2/3*d*	951	0.00%	1/3*d* plane	1/2*d* plane	−56.0362
Case 5	No. 2: 1/3*d*	951	0.00%	1/2*d* plane	2/3*d* plane	−33.2650
Case 6	No. 2: 2/3*d*	962	1.16%	1/2*d* plane	1/3*d* plane	−36.5967
Case 7	No. 1: 1/3*d*; No. 2: 1/3*d*	951	0.00%	2/3*d* plane	2/3*d* plane	−32.3376
Case 8	No. 1: 2/3*d*; No. 2: 2/3*d*	961	1.05%	1/3*d* plane	1/3*d* plane	−34.6353
Case 9	No. 1: 2/3*d*; No. 2: 1/3*d*	950	−0.11%	1/3*d* plane	2/3*d* plane	−32.5147
Case 10	No. 1: 1/3*d*; No. 2: 2/3*d*	961	1.05%	2/3*d* plane	1/3*d* plane	−40.5463

## Data Availability

The data used to support the findings of this study are available from the corresponding author upon request. The data are not publicly available due to privacy.
